# Exploratory Preoperative Risk Stratification of Uterine Mesenchymal Tumors in Postmenopausal Women with Complementary Transcriptomic Analysis

**DOI:** 10.3390/diagnostics16152409

**Published:** 2026-07-31

**Authors:** Xiaojie Wan, Tao Zhang, Jingyi Li, Zhimin Song, Fei Ruan, Jie Luo

**Affiliations:** Department of Obstetrics and Gynecology, Women’s Hospital, Zhejiang University School of Medicine, Hangzhou 310006, China; 5518006@zju.edu.cn (X.W.); changtao@zju.edu.cn (T.Z.); 06yxsyljy@zju.edu.cn (J.L.); songzhimin@zju.edu.cn (Z.S.)

**Keywords:** uterine mesenchymal tumors, uterine leiomyoma, postmenopausal women, malignancy risk stratification, transcriptomic profiling, cell cycle

## Abstract

**Background:** Preoperative differentiation between uterine mesenchymal tumors and benign uterine fibroids remains challenging in postmenopausal women. This study aimed to develop an exploratory risk-stratification model for uterine mesenchymal tumors and to characterize complementary molecular features of uterine leiomyosarcoma using a public transcriptomic dataset. **Methods:** A retrospective case–control study was conducted in postmenopausal patients with uterine masses who underwent surgery between 2011 and 2021. A total of 23 uterine mesenchymal tumor cases and 92 frequency-matched fibroid controls were included. Clinical variables were analyzed using univariable and multivariable logistic regression to develop an exploratory risk-stratification model. Model performance was evaluated using ROC analysis, calibration analysis, decision curve analysis, and confusion matrix assessment. Exploratory transcriptomic analysis was performed using the GEO dataset GSE64763 to characterize molecular differences between uterine leiomyosarcoma and fibroid tissues. **Results:** Pelvic pressure, abnormal uterine bleeding, and tumor diameter were independently associated with uterine mesenchymal tumors. The model achieved an apparent AUC of 0.859 and an optimism-corrected AUC of 0.846 after 1000 bootstrap resamples. Exploratory transcriptomic analysis revealed distinct expression patterns and upregulation of proliferation-associated genes, including CCNB1, BUB1B, PRC1, TOP2A, and FOXM1, with enrichment of cell cycle–related pathways. **Conclusions:** An exploratory model based on routinely available preoperative variables demonstrated preliminary discriminatory ability within the development cohort. Independent external validation in representative prospective cohorts is required before any assessment of clinical utility.

## 1. Introduction

Uterine fibroids are the most common benign smooth muscle tumors in women and are highly prevalent in both reproductive-age and perimenopausal populations [1,2]. Although most fibroids tend to regress after menopause because of hormonal changes, some postmenopausal women continue to present with persistent or enlarging uterine masses [3,4]. In this clinical setting, differentiating benign leiomyomas from malignant or potentially malignant uterine mesenchymal tumors remains a major diagnostic challenge.

Uterine sarcoma is a rare but highly aggressive gynecologic malignancy, accounting for approximately 3–5% of uterine tumors [5]. Among these, uterine leiomyosarcoma is the most common histological subtype [6]. Because uterine sarcomas often present with nonspecific symptoms and imaging findings that overlap substantially with benign fibroids, accurate preoperative diagnosis remains difficult [7,8]. Common manifestations, including abnormal uterine bleeding, pelvic pressure, abdominal discomfort, and enlarging uterine masses, lack sufficient specificity for reliable discrimination [9]. Delayed diagnosis may result in inappropriate surgical management, including morcellation, which has been associated with tumor dissemination and poor prognosis [10,11]. In clinical practice, the differential diagnosis also includes uterine mesenchymal tumors with uncertain or low malignant potential. In this study, uterine mesenchymal tumors therefore refer to the malignant and potentially malignant mesenchymal lesions included in the clinical cohort.

Postmenopausal status has been recognized as an important clinical risk factor for uterine sarcoma [12]. Previous studies have shown that the prevalence of unexpected uterine sarcoma increases with age, particularly in women older than 50 years [13]. Several studies have explored preoperative clinical indicators associated with uterine sarcoma, including tumor size, lactate dehydrogenase (LDH), abnormal uterine bleeding, and imaging characteristics [12,14]. However, existing evidence remains inconsistent, and reliable preoperative risk-stratification strategies for postmenopausal patients are still lacking. In addition to clinical heterogeneity, uterine sarcoma exhibits distinct molecular characteristics compared with benign fibroids, particularly in pathways related to cell-cycle progression and proliferative activity [15]. Transcriptomic analysis may therefore provide complementary biological context for clinical risk stratification of uterine mesenchymal tumors [16].

**Figure 1:**
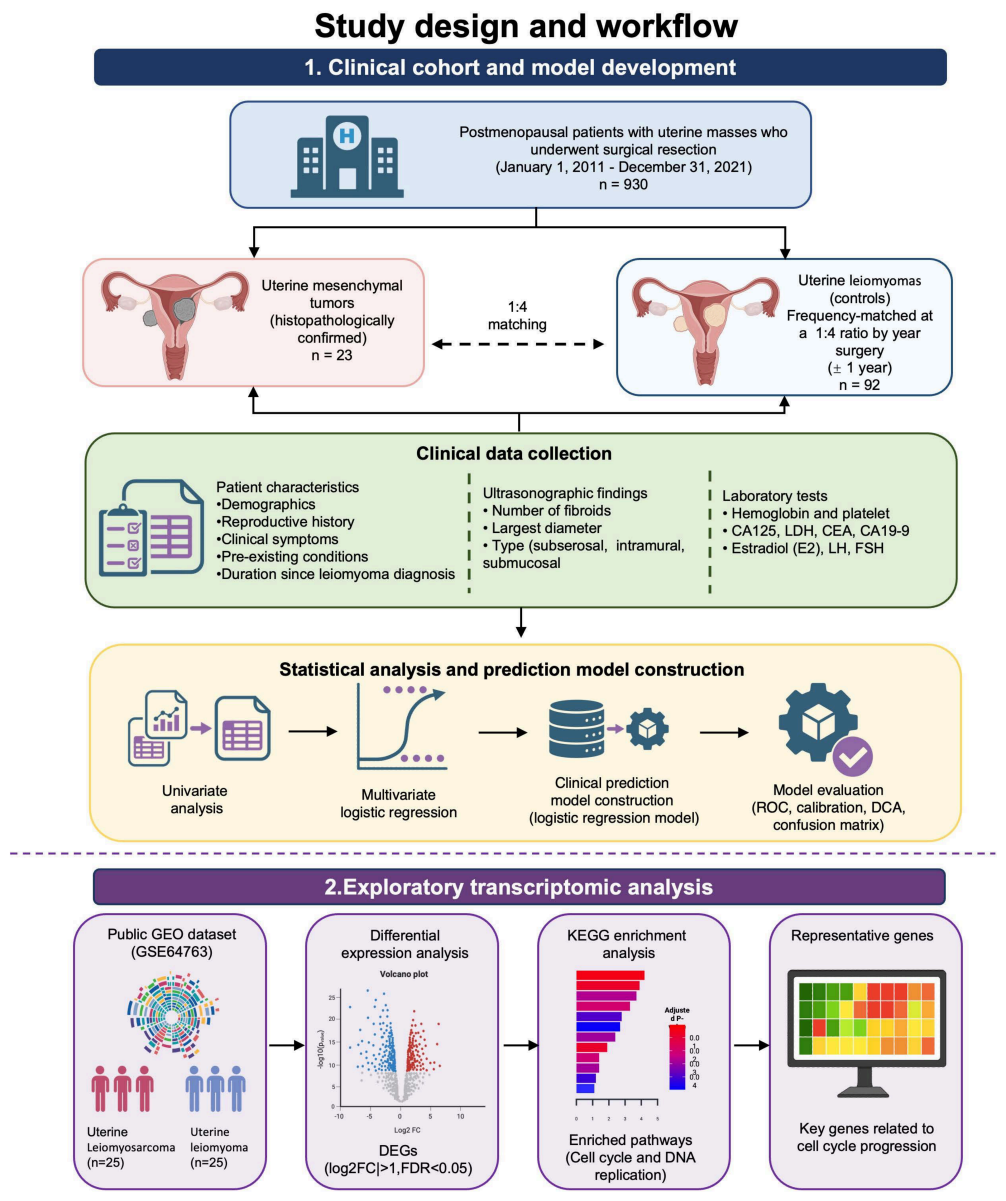
Study design and workflow.

Therefore, as summarized in Figure 1, we retrospectively analyzed postmenopausal women who underwent surgery for uterine masses and developed an exploratory multivariable risk-stratification model based on preoperative factors. Model performance was assessed using receiver operating characteristic (ROC) curve analysis, calibration analysis, decision curve analysis, and confusion matrix analysis. In parallel, a public Gene Expression Omnibus (GEO) dataset was analyzed to characterize differentially expressed genes and enriched pathways in uterine leiomyosarcoma compared with uterine fibroids.

## 2. Materials and Methods

### 2.1. Study Design and Patient Selection

As illustrated in Figure 1, this retrospective case–control study was conducted at the Women’s Hospital, Zhejiang University School of Medicine. The study was approved by the Institutional Ethics Committee (approval number: IRB-20250105-R, approval date: 18 February 2025). Postmenopausal women who underwent surgical resection for uterine masses between 1 January 2011 and 31 December 2021 were retrospectively screened. Postmenopausal status was defined as natural menopause for more than 12 months. Among 930 eligible patients, 23 patients with malignant or potentially malignant uterine mesenchymal tumors (hereafter referred to as uterine mesenchymal tumors) were identified; their histological composition is summarized in Supplementary Table S1. A 1:4 frequency-matching strategy based on date of surgery (±1 year) was used to select postmenopausal patients with uterine fibroids as controls to reduce potential temporal bias in clinical practice. Patients with other gynecological malignancies, incomplete postoperative pathological diagnoses, or insufficient clinical records were excluded. Atypical myometrial lesions, including smooth muscle tumors of uncertain malignant potential, were retained according to the postoperative pathological diagnosis. Because the case-to-control ratio was determined by frequency matching, the proportion of uterine mesenchymal tumors in this cohort should not be interpreted as reflecting its prevalence in routine clinical practice.

### 2.2. Clinical Data Collection

Clinical information was retrospectively collected from electronic medical records. Collected variables included demographic characteristics, reproductive history, clinical symptoms, pre-existing medical conditions, and duration since leiomyoma diagnosis. Available ultrasonographic variables were limited to the number of uterine masses, largest tumor diameter, and lesion location (subserosal, intramural, or submucosal). Detailed imaging characteristics, including lesion heterogeneity, irregular margins, necrosis, cystic degeneration, vascularity, rapid growth, and MRI findings, were not consistently documented and therefore could not be included in the analysis. Laboratory parameters included hemoglobin, platelet count, carbohydrate antigen 125 (CA125), LDH, carcinoembryonic antigen (CEA), carbohydrate antigen 19-9 (CA19-9), estradiol (E2), luteinizing hormone (LH), and follicle-stimulating hormone (FSH).

### 2.3. Exploratory Multivariable Logistic Regression Model Development and Performance Assessment

Exploratory multivariable logistic regression modeling was performed using Python 3.12.11 in Visual Studio Code 1.130.0. Data processing and model construction were conducted using pandas, numpy, scipy, statsmodels, scikit-learn, matplotlib, and seaborn libraries. Univariable logistic regression analysis was initially performed to identify clinical variables potentially associated with uterine mesenchymal tumors. Given the limited number of uterine mesenchymal tumor cases, multivariable model development was intentionally restricted to a small number of candidate predictors to reduce the risk of overfitting. Variable selection was based on clinical relevance, univariable regression results, data completeness, and model parsimony. Variables selected for multivariable analysis were entered simultaneously using the enter method. The final model included three predictors, corresponding to approximately 7.7 events per variable based on 23 events. Odds ratios (ORs) and 95% confidence intervals (CIs) were calculated. Continuous variables, including tumor diameter and LDH, were analyzed as continuous variables without categorization. Multicollinearity was assessed using variance inflation factors and tolerance values. L2-penalized logistic regression was performed as a sensitivity analysis, and internal validation was conducted using 1000 bootstrap resamples. The fitted probabilities generated by the exploratory model were used only for within-sample risk stratification and visualization and were not intended to estimate absolute clinical risk. Patients were ranked according to these fitted probabilities and classified into low-risk (<0.080), intermediate-risk (0.080–<0.168), and high-risk (≥0.168) groups for visualization. Model discrimination performance was evaluated using ROC curve analysis and area under the curve (AUC). Calibration performance was assessed using calibration curve analysis by comparing predicted and observed probabilities. Decision curve analysis was performed as an exploratory within-sample assessment of net benefit, and confusion matrix analysis was performed to assess classification performance at the optimal cutoff value. The optimal cutoff was determined using the Youden index. A sensitivity-prioritized cutoff of 0.080 was additionally evaluated, whereas 0.168 represented the Youden-derived cutoff.

### 2.4. Transcriptomic Data Analysis

Exploratory transcriptomic analysis was performed using the public GEO dataset GSE64763, comprising 25 uterine leiomyosarcoma and 25 uterine fibroid samples; 29 normal-myometrium samples were excluded from the prespecified comparison. The normalized series matrix was analyzed on its existing log2-transformed expression scale, and probe IDs were annotated using the GPL571 platform annotation. Principal component analysis was performed using scikit-learn after standardization of gene-expression values, and group separation was assessed using PERMANOVA based on Euclidean distances with 999 permutations. Differential expression analysis was conducted at the probe level using Welch’s *t*-test implemented in SciPy, followed by Benjamini–Hochberg correction. Probes with adjusted *p* < 0.05 and |log2 fold change| > 1 were considered differentially expressed. Kyoto Encyclopedia of Genes and Genomes (KEGG) pathway enrichment analysis was performed using the top 150 upregulated genes ranked by the t statistic through Enrichr in gseapy with the KEGG_2021_Human library. Heatmaps and box plots were generated using Matplotlib 3.10.9 and Seaborn 0.13.2. Detailed dataset characteristics and preprocessing procedures are provided in Supplementary Table S6.

### 2.5. Statistical Analysis

The distribution of continuous variables was assessed using the Shapiro–Wilk test. Continuous variables were summarized as median with interquartile range (IQR), and between-group comparisons were performed using the Mann–Whitney U test, chi-square test, or Fisher’s exact test, as appropriate. Missingness was summarized overall and by study group, with between-group differences assessed using Fisher’s exact test. Model-specific complete-case analysis was applied according to the predictors included in each model, and details of missing data are provided in Supplementary Table S2. Logistic regression was used for model development. Model comparison, penalized-regression sensitivity analysis, and bootstrap internal validation were performed as detailed in Supplementary Table S4. The optimal cutoff was determined using the Youden index, and a sensitivity-prioritized cutoff was also evaluated. All tests were two-sided, with *p* < 0.05 considered statistically significant. The sample size for a future validation study was estimated using a two-sided one-sample test of the AUC based on the Hanley–McNeil variance approximation. Assuming an expected AUC of 0.846, a reference AUC of 0.70, an α of 0.05, 80% power, and a 1:4 case-to-control ratio, the estimated minimum sample size was 165 participants.

## 3. Results

### 3.1. Clinical Characteristics of the Study Cohort

The baseline clinical characteristics of patients with uterine mesenchymal tumors and uterine fibroids are summarized in Table 1. No significant differences were observed between the two groups in age (56.00 [53.00–63.00] vs. 56.00 [53.75–63.00] years, *p* = 0.944) or body mass index (BMI) (23.42 [21.63–24.22] vs. 24.09 [21.75–26.25] kg/m^2^, *p* = 0.354). Patients with uterine mesenchymal tumors presented with significantly larger tumor diameters than those with uterine fibroids (7.60 [6.55–8.70] vs. 4.40 [2.38–6.33] cm, *p* < 0.001). Serum LDH levels were higher in the uterine mesenchymal tumor group, although the difference did not reach statistical significance (198.00 [173.00–265.75] vs. 185.00 [174.00–204.00] U/L, *p* = 0.134). Hemoglobin levels were slightly lower in patients with uterine mesenchymal tumors (130.00 [122.50–135.00] vs. 132.50 [128.00–140.00] g/L, *p* = 0.032). Regarding clinical symptoms, pelvic pressure was significantly more frequent in the uterine mesenchymal tumor group than in the fibroid group (39.1% vs. 7.6%, *p* < 0.001). Similarly, abnormal uterine bleeding was more commonly observed in patients with uterine mesenchymal tumors (47.8% vs. 22.8%, *p* = 0.033). No significant difference was observed in the number of leiomyomas between groups (*p* = 0.319), although multiple lesions were more frequently observed in patients with uterine mesenchymal tumors (52.2% vs. 38.0%). LDH was missing in 14 of 115 patients (12.2%), with similar proportions in the fibroid and uterine mesenchymal tumor groups (12.0% vs. 13.0%, *p* = 1.000). Complete-case analysis retained 101 patients; detailed missing-data patterns are shown in Supplementary Table S2.

### 3.2. Development of the Exploratory Multivariable Logistic Regression Model

**Figure 2:**
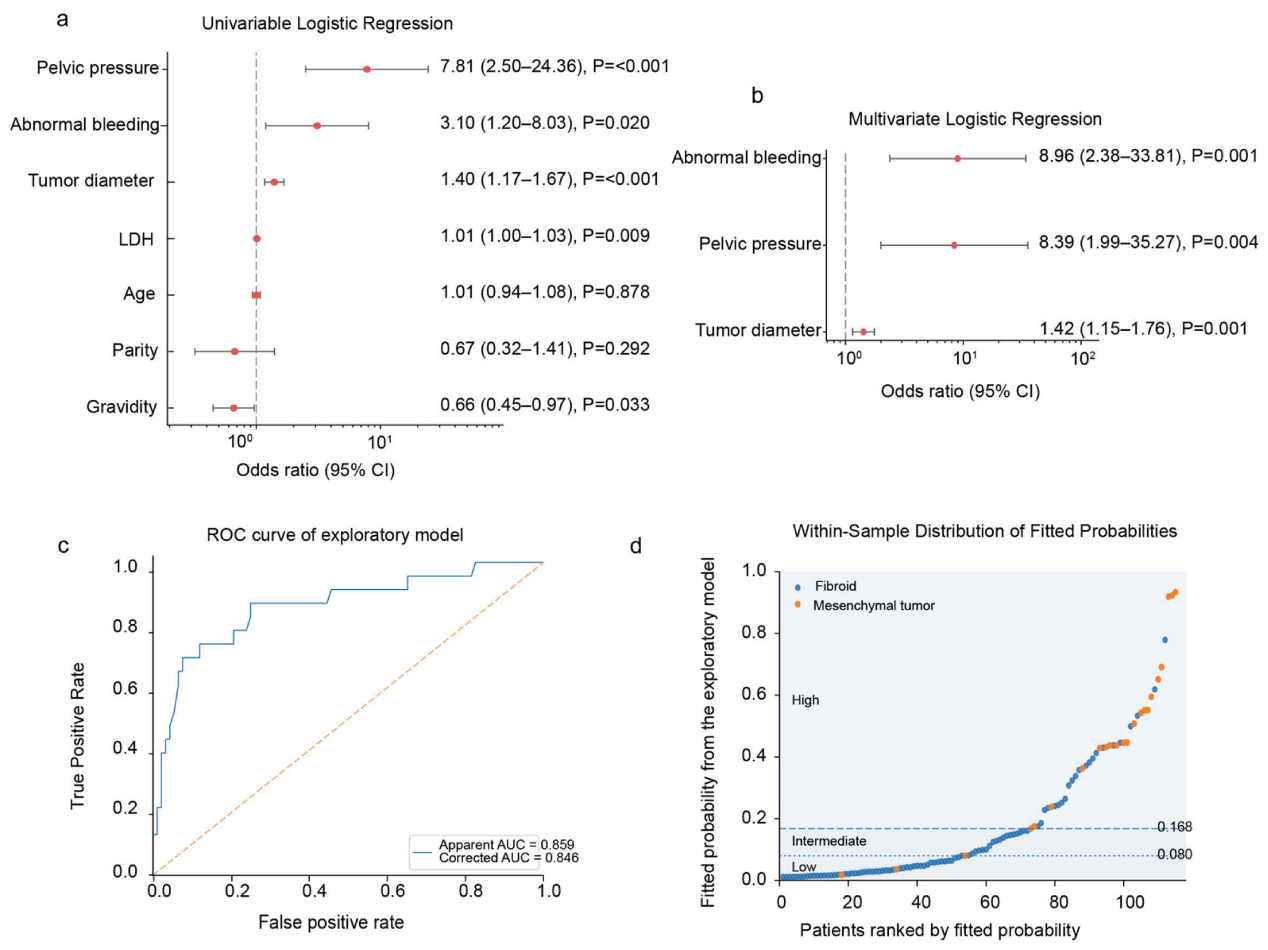
Exploratory multivariable logistic regression modeling and within-sample risk stratification. (**a**) Univariable and (**b**) multivariable logistic regression analyses. (**c**) ROC curve with apparent and bootstrap-corrected AUCs. (**d**) Within-sample distribution of fitted probabilities with cutoffs of 0.080 and 0.168.

To identify preoperative factors associated with uterine mesenchymal tumors, univariable logistic regression analysis was initially performed. Pelvic pressure (OR 7.81, 95% CI 2.50–24.36, *p* < 0.001), abnormal uterine bleeding (OR 3.10, 95% CI 1.20–8.03, *p* = 0.020), tumor diameter (OR 1.40, 95% CI 1.17–1.67, *p* < 0.001), and LDH (OR 1.014, 95% CI 1.004–1.025, *p* = 0.009) were significantly associated with uterine mesenchymal tumors. Gravidity was also associated with uterine mesenchymal tumors (OR 0.66, 95% CI 0.45–0.97, *p* = 0.033), whereas age and parity were not significant (Figure 2a). Multivariable logistic regression analysis further identified abnormal uterine bleeding (OR 8.96, 95% CI 2.38–33.81, *p* = 0.001), pelvic pressure (OR 8.39, 95% CI 1.99–35.27, *p* = 0.004), and tumor diameter (OR 1.42, 95% CI 1.15–1.76, *p* = 0.001) as independent factors associated with uterine mesenchymal tumors (Figure 2b). Detailed regression results are shown in Supplementary Table S3. Adding LDH did not significantly improve model fit (likelihood-ratio *p* = 0.113; Supplementary Table S4). Based on these variables, an exploratory multivariable logistic regression model was developed. The final model equation was: logit(*p*) = −4.757 + 2.127 × pelvic pressure + 2.193 × abnormal uterine bleeding + 0.350 × tumor diameter, where pelvic pressure and abnormal uterine bleeding were coded as 1 when present and 0 when absent. The predicted probability of having a uterine mesenchymal tumor was calculated as *p* = 1/[1 + exp(−logit(*p*))]. In the development cohort, the model yielded an apparent AUC of 0.859 and an optimism-corrected AUC of 0.846 (Figure 2c). Within-sample distribution analysis based on the fitted probabilities showed a greater concentration of uterine mesenchymal tumor cases in the higher-probability region, whereas most uterine fibroid cases were distributed in the lower-probability region (Figure 2d). Given the limited number of uterine mesenchymal tumor cases, these findings should be interpreted cautiously and require validation in larger independent cohorts.

### 3.3. Exploratory Assessment of Model Performance

**Figure 3:**
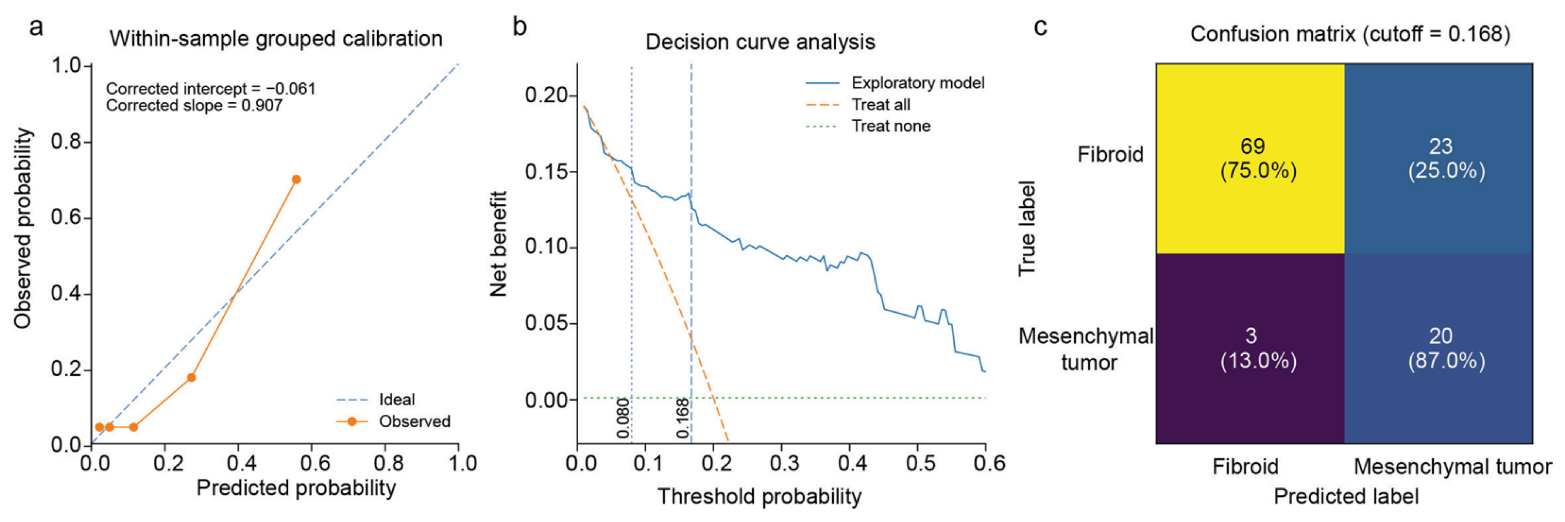
Exploratory assessment of model performance. (**a**) Calibration curve. (**b**) Decision curve analysis. (**c**) Confusion matrix at the cutoff of 0.168,cell colors represent patient counts, with lighter colors indicating higher values.

Within-sample calibration and decision curve analyses are presented in Figure 3a,b. However, because the case-to-control ratio was determined by frequency matching, these results reflect performance within the study sample and should not be interpreted as evidence of real-world calibration or clinical utility. At the Youden-derived cutoff of 0.168, the model identified 20 of 23 uterine mesenchymal tumor cases and 69 of 92 fibroid cases (Figure 3c). Three uterine mesenchymal tumor cases were misclassified, corresponding to a false-negative rate of 13.0%. A sensitivity-prioritized cutoff of 0.080 increased sensitivity to 91.3%, with a corresponding reduction in specificity to 54.3%. These thresholds are exploratory and require evaluation in clinically representative cohorts. Performance across additional selected probability thresholds is summarized in Supplementary Table S5.

### 3.4. Exploratory Transcriptomic Analysis

**Figure 4:**
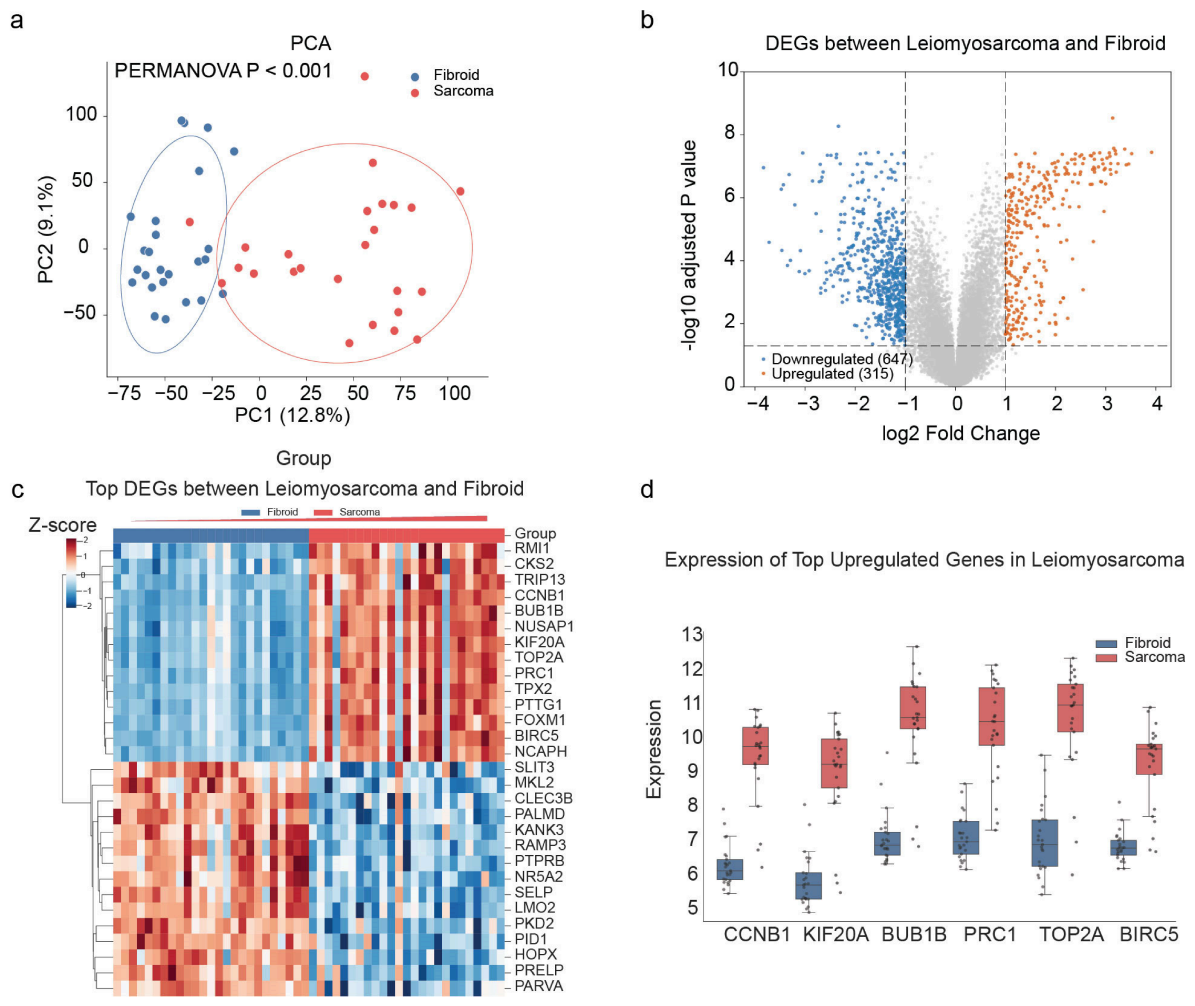
Exploratory transcriptomic analysis. (**a**) PCA of uterine leiomyosarcoma and uterine leiomyoma samples. (**b**) Volcano plot of differentially expressed probes, Grey dots indicate non-significant genes. (**c**) Heatmap of the top differentially expressed probes. (**d**) Expression levels of representative upregulated genes.

Exploratory transcriptomic analysis was performed using the public GEO dataset GSE64763 and included 25 uterine leiomyosarcoma and 25 uterine fibroid samples; 29 normal-myometrium samples were excluded from the prespecified comparison. Principal component analysis (PCA) demonstrated clear transcriptomic separation between uterine leiomyosarcoma and fibroid tissues (PERMANOVA *p* < 0.001), indicating distinct global gene expression patterns between groups (Figure 4a). Differential expression analysis identified substantial transcriptomic alterations in uterine leiomyosarcoma, including 315 upregulated and 647 downregulated probes (Figure 4b). Hierarchical clustering analysis further demonstrated robust segregation of leiomyosarcoma and fibroid samples based on the top differentially expressed genes (Figure 4c). Notably, multiple proliferation-related genes, including CCNB1, KIF20A, BUB1B, PRC1, TOP2A, and BIRC5, were significantly upregulated in uterine leiomyosarcoma compared with fibroid tissues (Figure 4d), supporting the highly proliferative biological characteristics of uterine mesenchymal tumors. Detailed cohort characteristics and preprocessing procedures are provided in Supplementary Table S6.

### 3.5. Cell Cycle–Related Transcriptomic Features of Uterine Leiomyosarcoma

**Figure 5:**
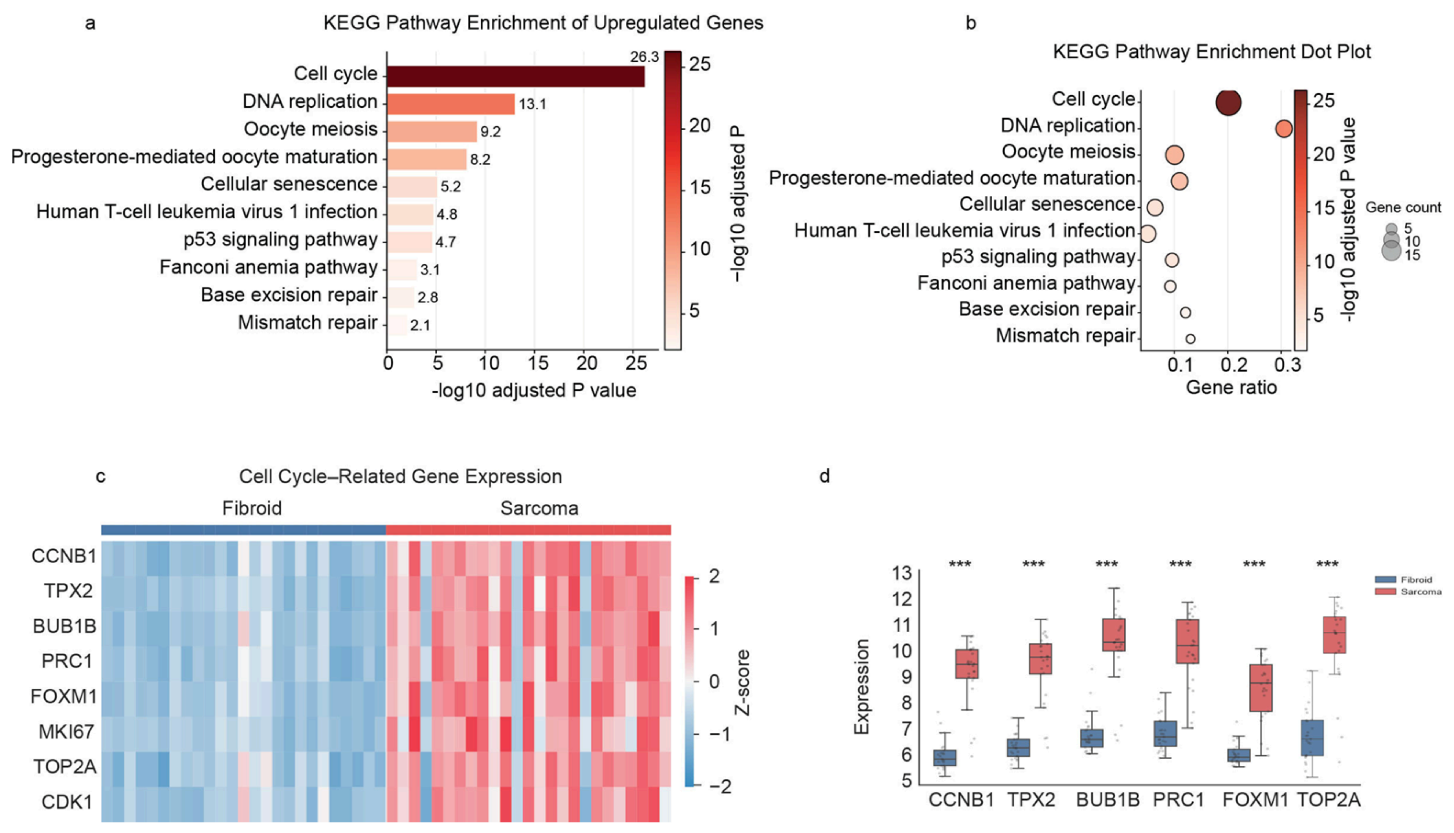
Cell cycle–related transcriptomic features. (**a**) KEGG pathway enrichment analysis. (**b**) KEGG enrichment dot plot. (**c**) Heatmap of representative cell cycle–related genes. (**d**) Expression of representative proliferation-associated genes (*** *p* < 0.001).

To further characterize the transcriptomic features associated with uterine leiomyosarcoma, KEGG pathway enrichment analysis was performed using the top 150 upregulated genes ranked by the t statistic. Cell cycle–related pathways were identified among the most significantly enriched biological processes, together with pathways associated with DNA replication, oocyte meiosis, progesterone-mediated oocyte maturation, and cellular senescence (Figure 5a,b). Heatmap analysis further demonstrated distinct expression patterns of representative cell cycle–related genes between uterine leiomyosarcoma and fibroid tissues (Figure 5c). In particular, multiple proliferation-associated genes, including CCNB1, TPX2, BUB1B, PRC1, FOXM1, and TOP2A, showed significantly increased expression in uterine leiomyosarcoma samples compared with fibroid tissues (all *p* < 0.001; Figure 5d), suggesting distinct proliferative transcriptomic features in uterine leiomyosarcoma.

## 4. Discussion

In the present study, we developed an exploratory risk-stratification model for differentiating uterine mesenchymal tumors from benign uterine fibroids in postmenopausal women and performed exploratory transcriptomic analysis to characterize the molecular features of uterine leiomyosarcoma. Uterine mesenchymal tumors are rare but potentially aggressive neoplasms, and their preoperative diagnosis is often challenging because of overlapping clinical manifestations with benign leiomyomas [17,18]. Focusing specifically on postmenopausal patients, a population with relatively increased malignancy risk, we identified several preoperative clinical factors associated with uterine mesenchymal tumors and showed that combining these variables yielded preliminary discriminative performance in the present cohort. In parallel, transcriptomic analysis revealed distinct proliferative and cell cycle–related molecular features in uterine leiomyosarcoma, providing complementary biological context rather than biological validation or mechanistic confirmation of the exploratory risk-stratification model.

Among the evaluated clinical variables, pelvic pressure, abnormal uterine bleeding, and tumor diameter were independently associated with uterine mesenchymal tumors in multivariable analysis. These findings are generally consistent with previous studies suggesting that symptomatic presentation and tumor burden may reflect more aggressive biological behavior in uterine sarcoma [12,19]. In particular, increasing tumor diameter has repeatedly been reported as an important clinical indicator for malignancy risk, although optimal cutoff values remain inconsistent across studies [20,21]. In the present study, tumor diameter was analyzed as a continuous variable rather than dichotomized using arbitrary thresholds, which may better preserve clinical information and reduce potential bias [22]. Based on these factors, we constructed a multivariable logistic regression model that showed preliminary within-sample discrimination, with an apparent AUC of 0.859 and an optimism-corrected AUC of 0.846. Additional calibration analysis, decision curve analysis, and confusion matrix analyses provided exploratory information on model performance but did not establish clinical utility. Importantly, the overall framework remained relatively simple and readily interpretable. Its reliance on routinely available preoperative variables supports further prospective evaluation without requiring complex imaging or highly specialized biomarkers.

Previous preoperative prediction models have combined clinical symptoms, serum biomarkers, and imaging features [23,24]. The present model was based on readily available clinical variables, but the absence of detailed imaging data prevented assessment of its incremental value beyond routine ultrasound. Ultrasound remains the first-line modality for uterine masses, although overlapping sonographic features limit its reliability in distinguishing benign from malignant lesions [25]. Tru-Cut biopsy may provide histological information in selected suspicious cases, but sampling error and intratumoral heterogeneity can reduce diagnostic accuracy [26]. Future studies should evaluate integrated clinical, laboratory, imaging, and biopsy-based approaches.

To further characterize the molecular features of uterine leiomyosarcoma, we performed exploratory transcriptomic analysis using the public GEO dataset GSE64763, which was originally generated to examine molecular profiles of uterine leiomyosarcoma, fibroids, and normal myometrium [27]. Global transcriptomic analysis demonstrated clear separation between uterine leiomyosarcoma and fibroid tissues, indicating substantial biological differences between the two entities. Differential expression analysis further identified multiple proliferation-associated genes that were significantly upregulated in uterine leiomyosarcoma, including CCNB1, BUB1B, PRC1, TOP2A, and FOXM1 [27,28]. In addition, KEGG enrichment analysis consistently highlighted pathways related to cell cycle progression, DNA replication, and mitotic regulation [29]. Although the present transcriptomic analysis was exploratory in nature and not intended to establish definitive molecular mechanisms, these findings suggest that the aggressive clinical behavior of uterine mesenchymal tumors may be associated with enhanced proliferative activity and dysregulated cell cycle programs. Because the transcriptomic analysis was performed in an independent public dataset rather than the clinical cohort, these findings should be interpreted as complementary biological context from a parallel exploratory investigation, rather than as biological validation or an integrated validation strategy for the exploratory clinical model.

Several limitations should also be acknowledged. This retrospective single-center study included only 23 uterine mesenchymal tumor cases, limiting statistical power and increasing the risk of overfitting. The frequency-matched case–control design also restricts interpretation of predicted probabilities and real-world clinical utility. At the Youden-derived cutoff, 3 of 23 uterine mesenchymal tumor cases were missed, indicating that the model cannot serve as a stand-alone rule-out tool. Missing laboratory data reduced the sample available for LDH-related analyses, and complete-case analysis may have introduced selection bias. Detailed imaging features were unavailable for many patients, limiting evaluation of their incremental predictive value. In addition, the transcriptomic analysis was based on a public leiomyosarcoma dataset with limited clinical metadata and should be regarded as exploratory. Therefore, these exploratory findings require confirmation in larger, multicenter prospective cohorts. A future validation study would require approximately 165 participants, including 33 uterine mesenchymal tumor cases and 132 fibroid controls, and should ideally include both premenopausal and postmenopausal women to assess generalizability across menopausal populations. Accordingly, neither the discrimination metrics nor the calibration and decision-curve analyses should be interpreted as evidence supporting immediate clinical implementation.

## 5. Conclusions

In summary, preoperative differentiation of uterine mesenchymal tumors from benign uterine leiomyomas remains challenging in postmenopausal women because of overlapping clinical manifestations. In this study, pelvic pressure, abnormal uterine bleeding, and tumor diameter were identified as factors associated with uterine mesenchymal tumors, and a readily interpretable exploratory risk-stratification model based on these variables showed preliminary discriminative performance in the present cohort. Furthermore, exploratory transcriptomic analysis revealed distinct proliferative and cell cycle–related molecular features in uterine leiomyosarcoma. Overall, the exploratory model provides preliminary information for the future development of preoperative risk-stratification approaches, while the transcriptomic analysis offers complementary biological context. Given the limited sample size, retrospective case–control design, incomplete imaging information, and lack of independent external validation, these findings should be considered exploratory and require confirmation in larger multicenter prospective cohorts before any assessment of clinical utility.

## Figures and Tables

**Table 1:** Baseline characteristics of patients with uterine mesenchymal tumors and uterine fibroids.

Variable	Fibroids	Mesenchymal Tumors	*p*-Value
Age (years)	56.00 [53.75–63.00]	56.00 [53.00–63.00]	0.944
BMI (kg/m^2^)	24.09 [21.75–26.25]	23.42 [21.63–24.22]	0.354
Tumor diameter (cm)	4.40 [2.38–6.33]	7.60 [6.55–8.70]	<0.001
LDH (U/L)	185.00 [174.00–204.00]	198.00 [173.00–265.75]	0.134
Hemoglobin (g/L)	132.50 [128.00–140.00]	130.00 [122.50–135.00]	0.032
Pelvic pressure			<0.001
No	85 (92.4%)	14 (60.9%)	
Yes	7 (7.6%)	9 (39.1%)	
Abnormal uterine bleeding			0.033
No	71 (77.2%)	12 (52.2%)	
Yes	21 (22.8%)	11 (47.8%)	
Number of leiomyomas			0.319
Single	57 (62.0%)	11 (47.8%)	
Multiple	35 (38.0%)	12 (52.2%)	

Data are presented as median [interquartile range] or *n* (%).

## Data Availability

The original contributions presented in this study are included in the article/Supplementary Materials. Further inquiries can be directed to the corresponding authors.

## Supplementary Materials

The following supporting information can be downloaded at https://www.mdpi.com/article/10.3390/diagnostics16152409/s1: Table S1: Histological composition of the 23 malignant or potentially malignant uterine mesenchymal tumors included in the clinical cohort; Table S2: Missing data by diagnostic group; Table S3: Univariable and multivariable logistic regression analyses of factors associated with uterine mesenchymal tumors; Table S4: Model development, multicollinearity assessment, sensitivity analysis, and bootstrap internal validation; Table S5: Within-sample classification performance at selected fitted-probability thresholds; Table S6: Characteristics and preprocessing of the public transcriptomic cohort.
