# Supplementary material for: Exploratory Preoperative Risk Stratification of Uterine Mesenchymal Tumors in Postmenopausal Women with Complementary Transcriptomic Analysis
Source: Diagnostics (Basel). 2026 Jul 31;16(15):2409. doi: 10.3390/diagnostics16152409 (PMC13465945; doi:10.3390/diagnostics16152409)
Supplement: Supplementary file 1 [file diagnostics-16-02409-s001.zip › diagnostics-4352871-supplementary.pdf]

Exploratory Preoperative Risk Stratification of Uterine Mesenchymal Tumors in Postmenopausal Women with Complementary Transcriptomic Analysis

Supporting Information

Supplementary Tables S1–S6

Contents

Supplementary Table S1. Histological composition of the 23 malignant or potentially malignant uterine mesenchymal tumors included in the clinical cohort

Supplementary Table S2. Missing data by diagnostic group

Supplementary Table S3. Univariable and multivariable logistic regression analyses of factors associated with uterine mesenchymal tumors

Supplementary Table S4. Model development, multicollinearity assessment, sensitivity analysis, and bootstrap internal validation

Supplementary Table S5. Within-sample classification performance at selected fitted-probability thresholds

Supplementary Table S6. Characteristics and preprocessing of the public transcriptomic cohort

Supplementary Table S1. Histological composition of the 23 malignant or potentially malignant uterine mesenchymal tumors included in the clinical cohort.

Histological subtype	n (%)
Uterine leiomyosarcoma	7 (30.4)
Uterine adenosarcoma	4 (17.4)
Endometrial stromal sarcoma	2 (8.7)
Smooth muscle tumor of uncertain malignant potential	2 (8.7)
Malignant inflammatory myofibroblastic tumor	1 (4.3)
Uterine tumor resembling ovarian sex cord tumor	1 (4.3)
Uterine mesenchymal tumor of low malignant potential	1 (4.3)
Unclassified uterine sarcoma	5 (21.7)
**Total**	**23 (100.0)**

Note. Uterine leiomyosarcoma included one myxoid leiomyosarcoma; endometrial stromal sarcoma included one low-grade endometrial stromal sarcoma. Cases without a specific histological subtype were categorized as unclassified uterine sarcoma according to the available pathological records.

Supplementary Table S2. Missing data by diagnostic group.

Variable	Overall, n (%)	Fibroid, n (%)	Uterine mesenchymal tumor, n (%)	P value
SCC	17 (14.8)	15 (16.3)	2 (8.7)	0.518
Endometrial thickness	15 (13.0)	10 (10.9)	5 (21.7)	0.177
LDH	14 (12.2)	11 (12.0)	3 (13.0)	1.000
CA19-9	10 (8.7)	10 (10.9)	0 (0.0)	0.208
E2	9 (7.8)	9 (9.8)	0 (0.0)	0.201
CEA	8 (7.0)	8 (8.7)	0 (0.0)	0.354
FSH	8 (7.0)	8 (8.7)	0 (0.0)	0.354
LH	8 (7.0)	8 (8.7)	0 (0.0)	0.354
CA125	4 (3.5)	4 (4.3)	0 (0.0)	0.582
Surgery type	3 (2.6)	3 (3.3)	0 (0.0)	1.000
Postoperative hemoglobin	1 (0.9)	1 (1.1)	0 (0.0)	1.000
Myoma type	1 (0.9)	1 (1.1)	0 (0.0)	1.000

Note. Values indicate the number and percentage of patients with missing data. P values compare missingness between the fibroid and uterine mesenchymal tumor groups.

Supplementary Table S3. Univariable and multivariable logistic regression analyses of factors associated with uterine mesenchymal tumors.

Section	Variable	N	Uterine mesenchymal tumor, n	Fibroid, n	Beta	SE	OR (95% CI)	P value
A. Univariable logistic regression	Pelvic pressure	115	23	92	2.055	0.581	7.81 (2.50–24.36)	<0.001
	Abnormal uterine bleeding	115	23	92	1.131	0.486	3.10 (1.20–8.03)	0.020
	Tumor diameter	115	23	92	0.336	0.091	1.40 (1.17–1.67)	<0.001
	LDH	101	20	81	0.014	0.005	1.014 (1.004–1.025)	0.009
	Age	115	23	92	0.006	0.036	1.01 (0.94–1.08)	0.878
	Parity	115	23	92	-0.397	0.377	0.67 (0.32–1.41)	0.292
	Gravidity	115	23	92	-0.416	0.195	0.66 (0.45–0.97)	0.033
B. Final three-variable multivariable model	Intercept	115	23	92	-4.757	0.949		<0.001
	Pelvic pressure	115	23	92	2.127	0.733	8.39 (1.99–35.27)	0.004
	Abnormal uterine bleeding	115	23	92	2.193	0.677	8.96 (2.38–33.81)	0.001
	Tumor diameter	115	23	92	0.35	0.109	1.42 (1.15–1.76)	0.001
C. Four-variable candidate model	Intercept	101	20	81	-6.298	1.54		<0.001
	Pelvic pressure	101	20	81	1.812	0.803	6.12 (1.27–29.53)	0.024
	Abnormal uterine bleeding	101	20	81	2.437	0.812	11.44 (2.33–56.14)	0.003
	Tumor diameter	101	20	81	0.342	0.121	1.41 (1.11–1.78)	0.005
	LDH	101	20	81	0.008	0.006	1.008 (0.997–1.020)	0.159

Note. CI, confidence interval; LDH, lactate dehydrogenase; OR, odds ratio; SE, standard error. Fibroid was coded as 0 and uterine mesenchymal tumor as 1. N denotes the number of complete cases included in each analysis. ORs for continuous predictors represent the change in odds per one-unit increase on the original measurement scale.

Supplementary Table S4. Model development, multicollinearity assessment, sensitivity analysis, and bootstrap internal validation.

Analysis	Measure / variable	Standard model result	Sensitivity-analysis / validation result	Additional statistic
Multicollinearity assessment	Pelvic pressure	N=101	VIF=1.414	Tolerance=0.707
	Abnormal uterine bleeding	N=101	VIF=1.133	Tolerance=0.883
	Tumor diameter	N=101	VIF=1.991	Tolerance=0.502
	LDH	N=101	VIF=1.665	Tolerance=0.601
Three- versus four-variable model comparison	Complete-case sample	101		
	Uterine mesenchymal tumor cases	20		
	Fibroid cases	81		
	AIC	74.871	74.360	-0.511
	BIC	85.331	87.435	2.104
	Apparent AUC	0.867	0.867	0.001
	Likelihood-ratio test		2.511	df=1; P=0.113
Penalized-regression sensitivity analysis	Pelvic pressure	N=115; β=2.127; OR=8.39 (1.99-35.27)	β=1.510; OR=4.53	Bootstrap 95% CI 1.67–12.01
	Abnormal uterine bleeding	N=115; β=2.193; OR=8.96 (2.38-33.81)	β=1.603; OR=4.97	Bootstrap 95% CI 2.01–12.75
	Tumor diameter	N=115; β=0.350; OR=1.42 (1.15-1.76)	β=0.318; OR=1.37	Bootstrap 95% CI 1.16–1.69
Bootstrap internal validation	AUC	Apparent=0.859	Optimism=0.013; Corrected=0.846	0.763–0.944
	Calibration intercept	Apparent=0.000	Optimism=0.061; Corrected=-0.061	-0.628–0.728
	Calibration slope	Apparent=1.000	Optimism=0.093; Corrected=0.907	0.493–1.377
	Brier score	Apparent=0.106	Optimism=-0.011; Corrected=0.117	0.081–0.157

Note. AIC, Akaike information criterion; AUC, area under the receiver operating characteristic curve; BIC, Bayesian information criterion; CI, confidence interval; LDH, lactate dehydrogenase; OR, odds ratio; VIF, variance inflation factor. Model comparison was performed using the same 101 complete cases. Penalized regression was conducted as a sensitivity analysis, and bootstrap internal validation used 1,000 resamples.

Supplementary Table S5. Within-sample classification performance at selected fitted-probability thresholds.

Threshold strategy	Cutoff	TP	FP	TN	FN	Sensitivity	Specificity	PPV	NPV	Accuracy	Balanced accuracy	F1 score	Youden index	Positive LR	Negative LR
Youden cutoff	0.168	20	23	69	3	0.870	0.750	0.465	0.958	0.774	0.810	0.606	0.620	3.478	0.174
Sensitivity-prioritized cutoff (target sensitivity ≥0.90)	0.080	21	42	50	2	0.913	0.543	0.333	0.962	0.617	0.728	0.488	0.457	2.000	0.160
Conventional cutoff	0.500	10	3	89	13	0.435	0.967	0.769	0.873	0.861	0.701	0.556	0.402	13.333	0.584

Note. FN, false negative; FP, false positive; LR, likelihood ratio; NPV, negative predictive value; PPV, positive predictive value; TN, true negative; TP, true positive. The sensitivity-prioritized cutoff was selected to achieve sensitivity of at least 0.90. These thresholds were evaluated within the development cohort and should not be interpreted as clinically validated decision thresholds.

Supplementary Table S6. Characteristics and preprocessing of the public transcriptomic cohort.

Characteristic	Description
Public dataset accession	GSE64763
Platform accession	GPL571
Platform	Affymetrix Human Genome U133A 2.0 Array
Technology	In situ oligonucleotide microarray
Organism	Homo sapiens
Data type	Gene-expression microarray
Total public samples	79
Fibroid samples	25
Leiomyosarcoma samples	25
Normal-myometrium samples	29
Samples included in the current analysis	50
Samples excluded from the current analysis	29
Primary comparison	Leiomyosarcoma versus fibroid
Tissue type	Human uterine tissue samples comprising fibroid, leiomyosarcoma, and normal myometrium
Expression-matrix file	GSE64763_series_matrix.txt
Platform-annotation file	GPL571-17391.txt
Expression scale	Normalized log2-transformed expression values
Expression-value range	3.58 to 14.75
Median expression value	7.19
Missing expression values	0
Preprocessing	The normalized GEO series matrix was analyzed on its existing log2 scale. Probe IDs were annotated with GPL571. Fibroid and leiomyosarcoma samples were compared at the probe level using Welch's t-test with Benjamini–Hochberg correction; normal-myometrium samples were excluded from the prespecified comparison.
Probe annotation	Probe IDs were mapped to gene symbols, gene titles, and Entrez Gene identifiers using GPL571.
Differential-expression method	Probe-level Welch's t-test
Multiple-testing correction	Benjamini–Hochberg false-discovery-rate correction
Differentially expressed probe definition	Adjusted P value < 0.05 and |log2 fold change| > 1
Clinical information available	GSM accession, sample title, tissue/source, and diagnostic group
Clinical information unavailable	Age, stage, grade, treatment, follow-up, survival, menopausal status, and other detailed patient-level variables.

Note. DEP, differentially expressed probe; GEO, Gene Expression Omnibus. The current differential-expression comparison included 25 leiomyosarcoma and 25 fibroid samples; 29 normal-myometrium samples were excluded from this comparison. This public transcriptomic dataset was analyzed as an independent exploratory cohort and was not used to validate the exploratory risk-stratification model.
